# African swine fever virus encodes for an E2-ubiquitin conjugating enzyme that is mono- and di-ubiquitinated and required for viral replication cycle

**DOI:** 10.1038/s41598-018-21872-2

**Published:** 2018-02-22

**Authors:** Ferdinando B. Freitas, Gonçalo Frouco, Carlos Martins, Fernando Ferreira

**Affiliations:** Centre for Interdisciplinary Research in Animal Health, Faculty of Veterinary Medicine, University of Lisbon, Lisbon, 1300–477 Portugal

## Abstract

African swine fever virus is the etiological agent of a contagious and fatal acute haemorrhagic viral disease for which there are no vaccines or therapeutic options. The ASFV encodes for a putative E2 ubiquitin conjugating enzyme (ORF I215L) that shows sequence homology with eukaryotic counterparts. In the present study, we showed that pI215L acts as an E2-ubiquitin like enzyme in a large range of pH values and temperatures, after short incubation times. Further experiments revealed that pI215L is polyubiquitinated instead of multi-mono-ubiquitinated and Cys85 residue plays an essential role in the transthioesterification reaction. In infected cells, I215L gene is transcribed from 2 hours post infection and immunoblot analysis confirmed that pI215L is expressed from 4 hpi. Immunofluorescence studies revealed that pI215L is recruited to viral factories from 8 hpi and a diffuse distribution pattern throughout the nucleus and cytoplasm. siRNA studies suggested that pI215L plays a critical role in the transcription of late viral genes and viral DNA replication. Altogether, our results emphasize the potential use of this enzyme as target for drug and vaccine development against ASF.

## Introduction

African swine fever (ASF) is a contagious haemorrhagic disease of domestic and wild suids, associated with mortality rates close to 100% and devastating socio-economic implications on affected regions^1,2^. Despite all the efforts applied to control the disease, the disease is solely controlled through the application of strict sanitary measures including among others, slaughtering of infected and exposed animals and trade restrictions^1,3^. Currently, ASF is endemic in most of sub-Saharan Africa, in Sardinia and since its introduction in Georgia via contaminated food in 2007, has been spreading through the Caucasus (Georgia, Armenia and Azerbaijan), Eastern Europe (Belarus, Moldova, Poland, Russia and Ukraine) and the Baltic countries (Estonia, Latvia and Lithuania)^4^. Caused by a large (≈200 nm) lipoprotein-enveloped, icosahedral, double-stranded DNA virus (170 to 190 kbp) and being the only member of *Asfarviridae* family, the African swine fever virus (ASFV) infects different species of soft ticks, wild and domestic pigs^5^. ASFV encodes for between 151 and 167 open reading frames (ORFs), with half of them lacking any known function^3,5,6^. As reported for other viruses^7,8^, ASFV must evade the cellular antiviral defenses and modulate gene expression to establish a productive infection, probably by disrupting the ubiquitination and SUMOylation status of host proteins. The ubiquitin pathway is a major cellular system consisting of enzymes that conjugate the 76-amino-acid protein tag ubiquitin to and deconjugate it from host target proteins for proteasomal degradation, thereby regulating signaling cascades and cell cycle. Interestingly, ASFV encodes for a putative ubiquitin-conjugating E2-like enzyme (pI215L, ASFV-UBCv1)^9^ found within the virion, suggesting that pI215L may be involved in the early steps of infection^10^. As previously described, ASFV-infected cells tightly regulate ubiquitin mRNA levels when compared to mock-infected cells, strengthening the idea that ASFV perverts the ubiquitin pathway to its own benefit^11^.

Although the exploitation of ubiquitin system by viruses is emerging as a central theme and several studies highlight the use of ubiquitin inhibitors as an antiviral approach^12^, few data is available on the ASFV ubiquitin-conjugating E2-like enzyme and its role during infection. Thus, this study aims to characterize the pI215L E2 ubiquitin-conjugating enzymatic activity *in vitro* and to evaluate the transcription pattern of the ASFV-I215L gene, as well as its expression and its distribution in infected cells. To better understand the importance of pI215L during infection, the transcription activity of early and late viral genes, the number of ASFV genomes and the viral progeny were analyzed and compared between I215L-knockdown cells and mock-transfected cells. Finally, the biological role of the pI215L in a cellular context was schematically illustrated, suggesting that pI215L can be a good candidate for the development of a vaccine against ASF or used as a target for antiviral therapy.

## Results

### pI215L acts as an E2-ubiquitin conjugating enzyme, binding one or two ubiquitin molecules at the cysteine 85, in an ATP- and Mg^2+^-dependent manner

**Figure 1:**
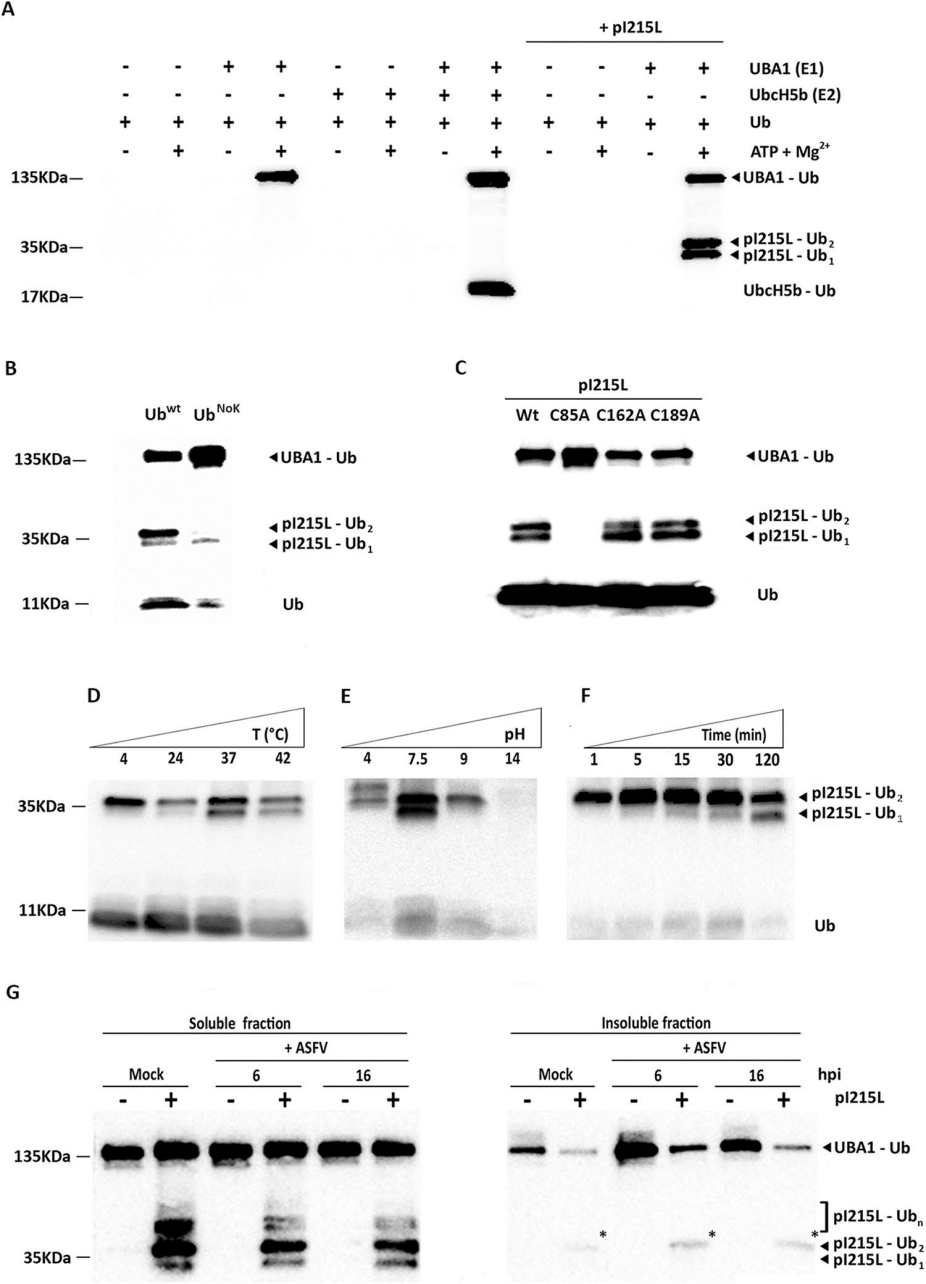
pI215l acts as an E2-ubiquitin conjugating enzyme. **(A)** Results from an *in vitro* ubiquitination assay showed that recombinant pI215L binds one or two ubiquitin molecules, in an ATP- and Mg^2+^-dependent manner, and in the presence of an E1 ubiquitin-activating enzyme (UBA1). Reaction mixtures were incubated 2 hours at 37 °C, quenched with a non-reducing protein loading buffer, and then subjected to polyacrylamide gel electrophoresis. **(B)** When the ubiquitination assay was performed using an ubiquitin that is mutated in all lysine residues (Ub^NoK^), the di-ubiquitinated forms of pI215L were not detected. **(C)** The residue cysteine-85 is essential for the E2-like activity of pI215L. Site-directed mutagenesis revealed that replacement of Cys-85 by an alanine residue led to an absence of ubiquitinated species, whereas the substitution of the Cys-162 or Cys-189 residue does not hamper the formation of ubiquitinated forms of pI215L. **(D)** pI215L forms thioester bonds with ubiquitin in a wide range of temperatures, although mono-ubiquitinated forms of pI215L were less detectable at 4 °C and 24 °C. **(E)** pI215L binds ubiquitin in a broad range of pH values, with mono-ubiquitinated forms only found at a pH value of 7.5. **(F)** Poly-ubiquitinated forms of I215L were detected after a short incubation period of 1 min, whereas the mono-ubiquitinated forms were detected later (5 min), showing increased concentrations in longer incubation times (e.g. 30, 60 minutes). **(G)** Mono- and poly-ubiquitinated forms of pI215L were mainly found in the Triton X-100-soluble fractions harvested at 6 and 16 hpi. In detergent-insoluble fractions, only the di-ubiquitinated form of pI215L was faintly detected (asterisks). Blots of Fig. 1(D to F) were cropped to improve clarity, full-length blots are presented in supplementary Figure S1. Fig. 1(G) is composed by two individual blots obtained from soluble and insoluble fractions.

Considering the sequence homology (49% identity) between the ASFV-pI215L (accession number: AJZ77128.1) and human E2-ubiquitin conjugating enzyme G2 (accession number: CBW46807.1), we aimed to confirm previous results which have shown that pI215L has the ability to bind ubiquitin, to determine the optimal conditions required for the formation of the thioester bond, to identify the cysteine residue of pI215L essential for the formation of the Ub~conjugates and to analyze the pI215L-ubiquitin conjugates forms present in detergent insoluble/soluble protein fractions collected from ASFV-infected cells. Immunoblot analysis showed that pI215L only binds to pre-activated ubiquitin (by an E1 enzyme, UBA1) and in the presence of ATP and Mg^2+^, similarly to the human E2-ubiquitin conjugating enzyme UbcH5b, used as control (Fig. 1A). Two distinct biotinylated-ubiquitin conjugates corresponding to mono-ubiquitinated (≈36 KDa, pI215L-Ub_1_) and di-ubiquitinated (≈47 KDa, pI215L-Ub_2_) species were detected. Since the upper band (pI215L-Ub_2_) can result from multi-ubiquitinated pI215L forms (two monoubiquitinations in two different cysteine residues) and/or from poly-ubiquitinated forms of pI215L (di-ubiquitination of one or more cysteine residues), the ubiquitin wild type was substituted by a commercial ubiquitin mutated in the seven acceptor lysine residues (Ub^NOK^), thus preventing ubiquitin chain elongation (polyubiquitination). The results obtained show a loss of the upper band when the Ub^NOK^ mutant replaces the ubiquitin wild type (Fig. 1B), indicating that pI215L has oligoubiquitin chains that contain only two ubiquitin molecules, not being multi-ubiquitinated. Taking in consideration these results, we next investigated if ubiquitin binds to pI215L using the same cysteine or not. Although cysteine residue at position 85 is conserved in all ASFV isolates and in eukaryotic E2-ubiquitin conjugating enzymes, and annotated as the putative catalytic residue of pI215L, its importance in ubiquitin ligation in unknown, as well as the Cys-162 and Cys-189 residues. In order to evaluate if these residues are responsible for the formation of mono- and di-ubiquitinated pI215L conjugates, three single point mutants were generated by site-directed mutagenesis: pI215L^C85A^, pI215L^C162A^ and pI215L^C189A^. Immunoblot results showed that replacement of the sulfur containing cysteine at position 85 by a nonpolar amino acid (alanine) totally inhibits the formation of ubiquitin pI215L conjugates contrasting with the pI215L^wt^ and the single point mutants: pI215L^C162A^ and pI215L^C189A^ (Fig. 1C). To investigate if a transesterification reaction mediates the transfer of ubiquitin between the E1-ubiquitin activating enzyme and cysteine-85 present at the active site of pI215L, reaction mixtures were incubated with the 2-mercaptoethanol (a reducing agent) and, as expected, the ubiquitin pI215L conjugates become lost after a short incubation period, indicating that ubiquitin binds to pI215L through a thioester bond (data not shown). Further experiments to characterize the binding activity of pI215L revealed that mono-ubiquitinated and di-ubiquitinated species were detectable in a wide range of temperatures, although their formation seems to be favored at 37 °C (Fig. 1D). When the reaction mixtures were incubated at different pH values, the E2-ubiquitin conjugating activity of pI215L was maximal at a pH value of 7.5, with the mono-ubiquitinated species not being detected at pH values below 4 or above 9. In addition, an upper band corresponding to poly-ubiquitinated forms was detected in acidic conditions and an almost complete absence of ubiquitin-conjugating activity was found at pH values of 14 (Fig. 1E). Interestingly, the formation of di-ubiquitinated conjugates was identified after short incubation times (e.g. 1 min), whereas mono-ubiquitinated pI215L forms were only detected at longer incubation times (Fig. 1F).

Finally, to better characterize the pI215L E2-ubiquitin conjugating activity, reaction mixtures were incubated with soluble and insoluble protein fractions prepared from mock infected and Ba71V-infected cells (6 and 16 hpi). Results revealed that pI215L has two distribution pools, with three species of pI215L-ubiquitin conjugates being detectable in the detergent soluble protein fraction (Fig. 1G) and only a faint band corresponding to di-ubiquitinated forms was observed in insoluble protein fractions (Fig. 1G, asterisks).

### ASFV-I215L gene encodes for a very early protein that localizes in viral factories and host cell nucleus

**Figure 2:**
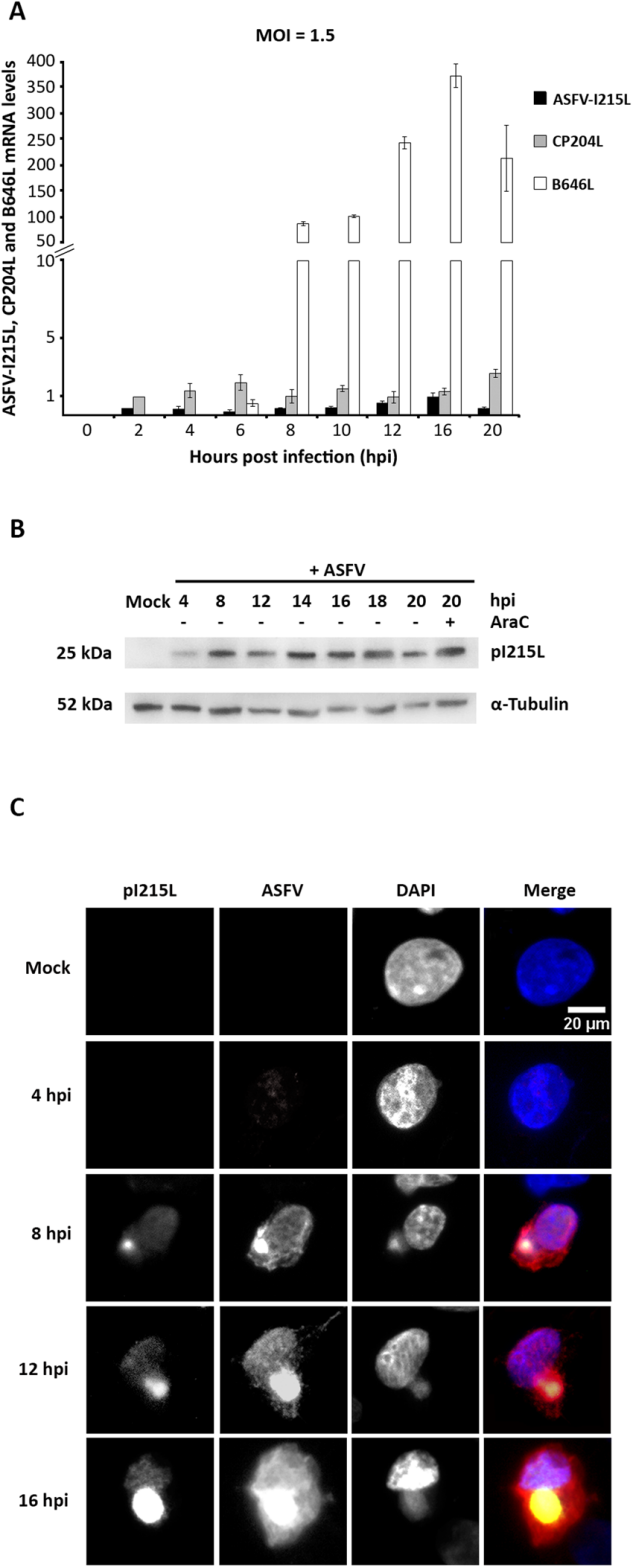
ORF I215L encodes an early viral protein that accumulates in viral factories. **(A)** I215L transcripts were detected from 2 hpi onwards showing a maximum peak at 16 hpi. The mRNA levels of CP204L (vp32) and B646L (vp72) were measured in parallel and used as controls of early and late viral gene expression, respectively. Results are shown as mean ± standard error by dividing the number of transcripts of each viral gene by the number of Cyclophilin A mRNA molecules (housekeeping gene), obtained from three independent experiments run in duplicate. **(B)** ASFV-I215L gene encodes for an early protein detectable from 4hpi onwards, with expressing levels unchanged by the AraC treatment. Vero cells infected with ASFV/Ba71V isolate (MOI of 5) and harvested at the indicated time points. The cytosine arabinoside treatment (AraC, 50 µg/ml) was performed after the initial viral adsorption period (1 hour) and cells were collected at 20 hpi. **(C)** Vero-infected cells (MOI = 2) were fixed (4, 8, 12 and 16 hpi), immunostained and analyzed by fluorescent microscopy. pI215L was detected from 8 hpi onwards, being recruited to viral factories, co-localizing with other viral proteins (e.g. pA104R and VP72, data not shown) and showing a faint distribution pattern in cell nucleus. In the merged images, pI215L, ASFV and DAPI staining is shown in green, red and blue, respectively. Representative images of at least three independent experiments are shown.

qPCR results revealed that ASFV-I215L gene is actively transcribed from 2hpi onwards (Fig. 2A), showing two transcription peaks at early and late infection time points (2 and 16 hpi), suggesting that pI215L is involved in different phases of viral life cycle. However, ASFV-I215L mRNA levels were much lower than the ones found in two viral genes that encode for structural proteins and were used as controls (the early CP204L viral gene and the late B646L viral gene). In order to ensure that normalized mRNA levels of three viral genes are comparable, only qPCR reactions with efficiency values ranged from 90 to 91% and showing R^2^ values > 0.987 were considered.

The immunoblot analysis showed that pI215L is detectable in ASFV-infected Vero cells from 4 hpi onwards (Fig. 2B), increasing its concentration throughout the infection, in accordance with I215L mRNA levels. As expected, pI215L was detected in whole extracts of infected cells exposed to cytosine arabinoside (AraC), an inhibitor of ASFV DNA replication and of late transcription phase, supporting that pI215L is an early viral protein (Fig. 2B). In parallel, immunostaining studies revealed that pI215L accumulates in viral cytoplasmic factories, colocalizes with other ASFV proteins and shows a diffuse nuclear pattern (Fig. 2C), from 8 hpi onwards.

### Knockdown of pI215L impairs viral infection

**Figure 3:**
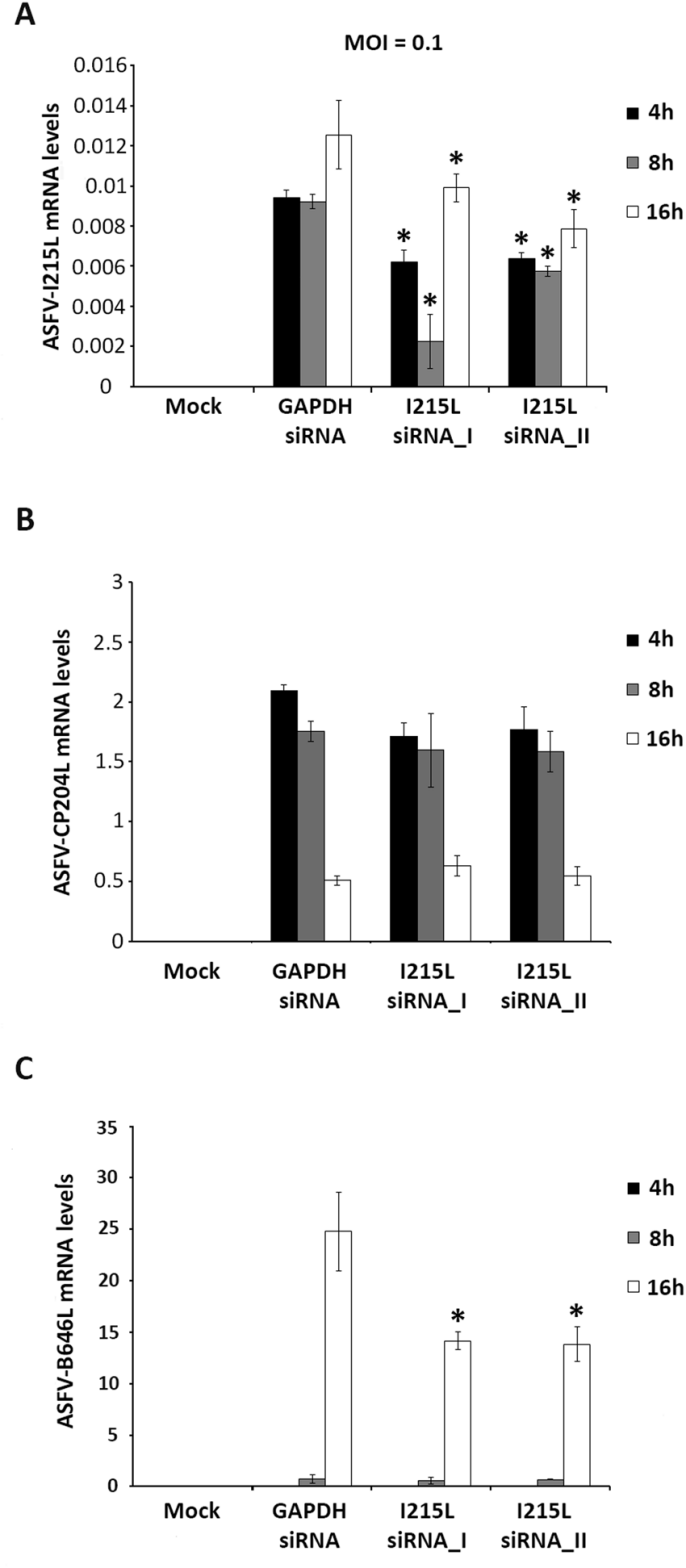
siRNAs targeting I215L disrupt late viral transcription. **(A)** siRNAs against I215L significantly reduced its mRNA levels at 4, 8 and 16 hpi in comparison to the infected control (p ≤ 0.05). I215L-depleted cells showed significantly lower mRNA levels of the ASFV B646L late gene (p ≤ 0.05) **(C)**, although the mRNA levels of the early viral gene CP204L (vp32) remained similar to the levels detected in control group **(B)**. Results are shown as average ± standard error (AVG ± S.E.), between the number of molecules of each viral transcript and the number of Cyclophilin A transcripts (housekeeping gene). Data were obtained from three independent experiments run in duplicate.

**Figure 4:**
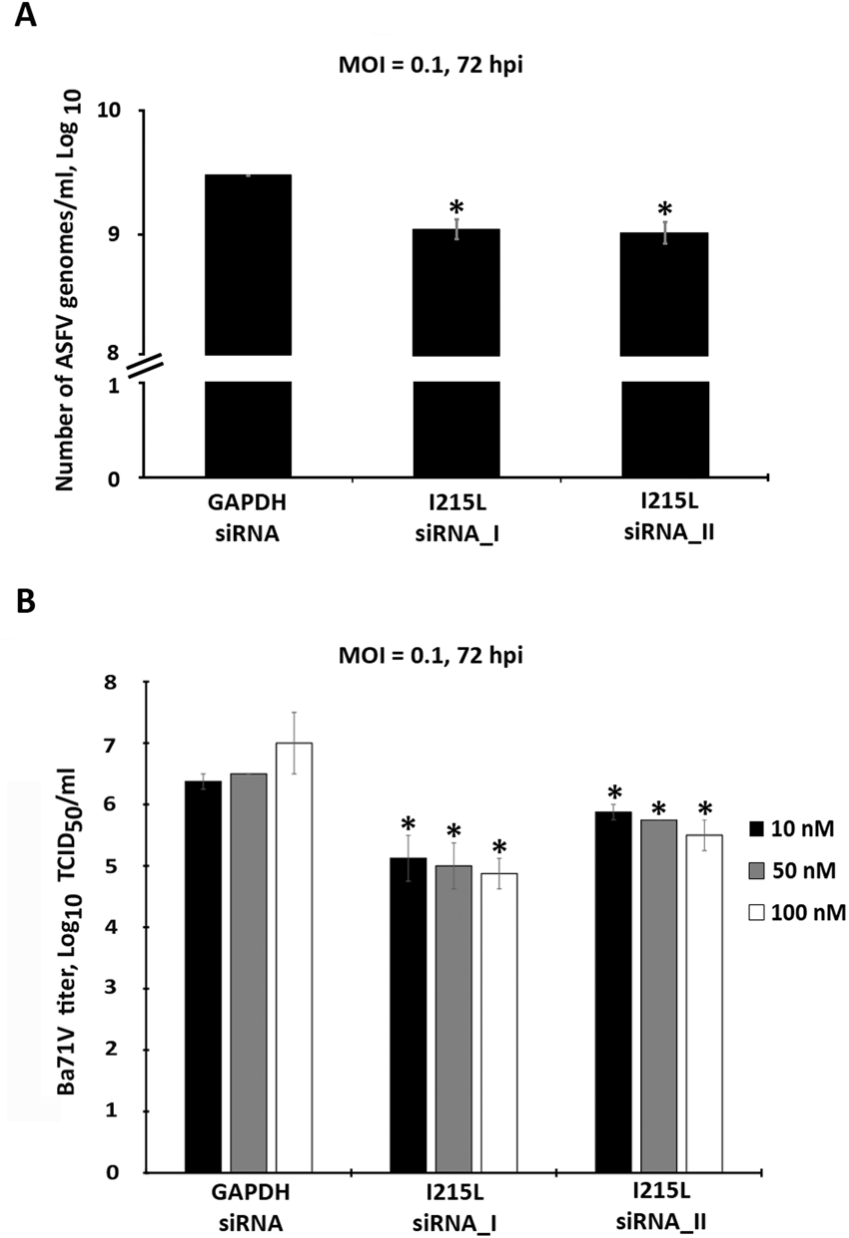
Knockdown of I215L mRNA levels inhibits ASFV DNA replication and progeny production. **(A)** I215L-depleted cells showed a decreased number of ASFV genomes [1.01 × 10^9^ genomes/ml for siRNA_I sequence (−65.83%) and 1.09 × 10^9^ genomes/ml for siRNA_II (−64.87%)] when compared to the control group (2.98 × 10^9^ genomes/ml, p ≤ 0.05). Results represent the mean of three independent experiments. **(B)** A statistically significant reduction in viral yield was observed in ASFV-infected Vero cells (MOI of 0.1) transfected with siRNAs against ASFV-I215L (100 nM), in comparison with the GAPDH siRNA-transfected infected cells (between −96.86% and −99.24%; 1 × 10^4.88^ and 1 × 10^5.50^ versus 1 × 10^7.00^ viral particles/ml; p ≤ 0.05), at 72 hpi. The virus yield of each supernatant was calculated from the average of three independent experiments. Error bars represent standard error (SE) of the mean values.

Considering the *in vitro* results, and the evidences that ASFV-I215L expression occurs during infection, siRNAs experiments were conducted to further explore the role of pI215L during infection. In order to avoid off-target effects and ensure the biological significance of the results, two siRNAs targeting I215L were used, showing significant knockdown efficiency at 4, 8 and 16 hpi (from −23% to −40%, Fig. 3A). The qPCR analysis also revealed that I215L-knockdown cells showed reduced mRNA levels of the late B646L viral gene (up to −57.1%, Fig. 3C), when compared to control cells (transfected with siRNA against the housekeeping GAPDH gene), whereas the transcriptional activity of the early CP204L viral gene was unaltered (Fig. 3B). In addition, pI215L seems to be involved in ASFV DNA replication, with depleted cells showing lower number of viral genomes (−65.83% for siRNA_1, −64.87% for siRNA_2) and lower viral progeny (from −68.37% to −99.24%) when compared with Vero cells transfected with siRNA against GAPDH (p ≤ 0.05, Fig. 4A and B).

## Discussion

For more than 20 years, studies on ASFV have identified the presence of a putative E2 Ubiquitin-conjugating enzyme^10,13–15^, the first to be described in eukaryotic viruses^14,15^. Nowadays, it is well known that several viruses modulate the ubiquitin-proteasome system of cells, through different mechanisms, as encoding ubiquitin-related enzymes^12,16^. However, so far the role of the ubiquitination machinery in ASFV infection remains poorly understood, in particular, regarding the function of the viral E2-like protein (pI215L). In this study, we showed that pI215L has the capacity to bind one or two ubiquitin molecules pre-activated by an E1 ubiquitin-activating enzyme, reinforcing the hypothesis that pI215L acts as an E2-like enzyme^9^. This scenario is further supported by the loss of a thioester bond between the carboxyl-terminal of ubiquitin molecules and the conserved catalytic residue of pI215L identified by mutagenesis analysis (Cys 85), when the reducing agent 2-mercaptoethanol was added. In addition, the need of ATP and Mg^2+^ as cofactors, mimics the requirements of other E2 ubiquitin-conjugating enzymes^17,18^, strengthening the idea that pI215L acts as an E2 ubiquitin-conjugating enzyme.

Moreover, both ubiquitinated forms of pI215L were detected under a wide range of pH values (4 to 9), suggesting that pI215L found in the viral particles^10^ may remain catalytically active during the cell entry process, which occurs via a low-pH-dependent endosomal pathway^19,20^. This catalytic feature may also contribute to ensure the E2-like activity of pI215L in the midgut epithelial cells of the tick *Ornithodoros* spp., where the pH levels are lower than 7^21^. In parallel, pI215L-ubiquitin conjugates were also observed under a broad range of temperatures (4 °C to 42 °C), further suggesting that pI215L is active in the disease’s vector and in infected animals^22^. Interestingly, the di-ubiquitinated species were detected earlier (after 1 min of incubation) and in larger amounts than mono-ubiquitinated forms of pI215L (after 5–10 minutes). Although the monoubiquitination is well documented for E2 enzymes^23,24^, the formation of di-ubiquitinated forms was recently reported in E2 ubiquitin-conjugating enzymes^25^. In this last scenario, the ubiquitin chain pre-generated in the E2 active site may be transferred to a specific E3 ubiquitin ligase and then to the target protein^26^ or be related to a mechanism of E2 autoregulation that may lead to its degradation in the proteasome^27^. Moreover, the higher amounts of mono- and di-ubiquitinated forms detected with detergent-soluble protein fractions, as well as poly-ubiquitinated species, suggest that, in cellular context, pI215L may participate in distinct regulation mechanisms, since the ability to generate diverse substrate-ubiquitin structures is essential to target different host/viral proteins. Indeed, it is reported that monoubiquitination of several nuclear proteins modulates DNA repair and cellular gene expression^28,29^, whereas the polyubiquitination of a target protein occurs via K48 of ubiquitin can lead to protein degradation through the 26 S proteasome pathway or activated phosphatases^30,31^, or by endocytosis if polyubiquitination occurs via K63 residue of ubiquitin. Also noticeable is the distinct pool of di-ubiquitinated forms detected in detergent-insoluble extracts, probably caused due to the pI215L binding affinity to host proteins containing an ARID DNA-binding domain^11^. In non-infected cells, a stronger band of pI215L polyubiquitinated forms was detected and this result may be due to the absence of other viral proteins, which are acceptors of ubiquitin via pI125L and/or host proteins that are ubiquitinated by pI215L during infection course. However, we cannot exclude that non-infected cells may recognize the pI215L as a foreign protein, promoting its degradation by the host ubiquitin proteasome pathway.

**Figure 5:**
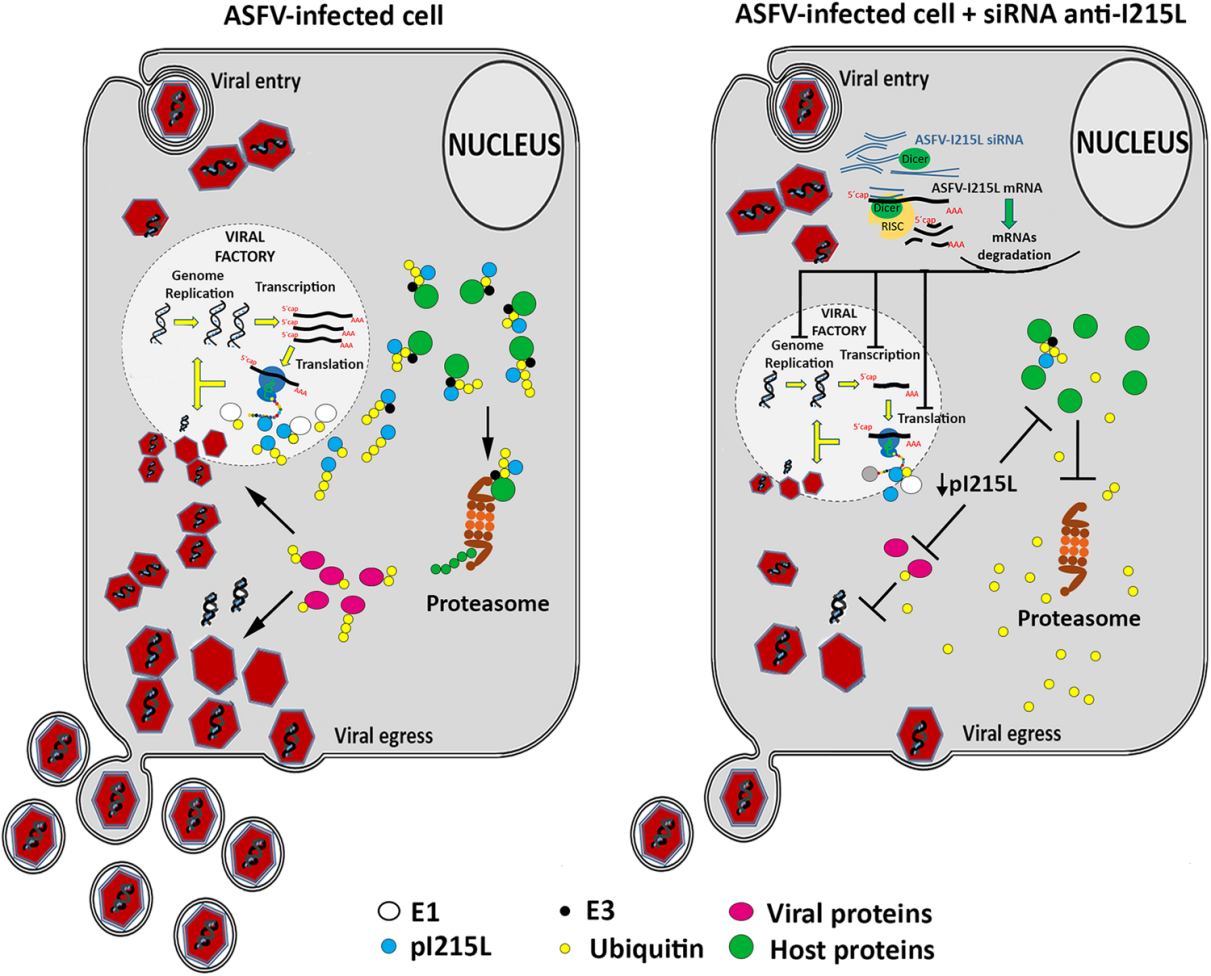
Proposed working model of the role of pI215L during ASFV infection. Once ASFV enters the host cell, different host mechanisms are subverted in other to generate a productive infection. By encoding an E2 ubiquitin-conjugating enzyme (pI215L), ASFV hijacks the cellular ubiquitin-proteasome system modulating the function and subcellular localization of host proteins and its own proteins. By controlling the ubiquitination status of the cellular proteins, viruses are able to evade host antiviral responses by targeting proteins to proteasomal degradation and to modulate the activity of viral proteins in different mechanisms. Our model suggests that by downregulating I215L expression, a reduction in the abundance of ubiquitin-tagged proteins occurs and consequently causes an inhibition of several crucial viral processes (e.g. genome replication, gene transcription, translation, egress), as well as host pathways (e.g. antiviral immune response, apoptosis).

Results obtained from the ASFV-infected Vero cells revealed that I215L viral gene is transcribed throughout infection, showing two transcription peaks (at 2 and 16 hpi), suggesting that pI215L may be required at different stages of the viral life cycle (e.g. viral transcription, genome replication and viral egress), as reported for other viruses^32^. As expected, the pI215L was detected throughout infection (from 4 hpi to 20 hpi), even in the presence of AraC, proving that pI215L is an early viral protein and corroborating the idea that ubiquitin expression must be tightly regulated during ASFV infection^33^. Immunolocalization studies revealed that pI215L is recruited to viral factories, strengthening the hypothesis that this viral E2 ubiquitin-conjugating enzyme is involved in viral transcription and/or DNA replication, while its diffuse distribution throughout the cytoplasm may be related to its role in ubiquitination of viral proteins and/or host proteins involved in nuclear functions (e.g. antiviral responses, DNA damage responses). Finally, results from siRNA experiments disclosed that pI215L is involved in the late viral transcription with pI215L-knockdown cells showing a lower number of B646L transcripts, while the mRNA levels of the early viral gene CP204L remained unchanged in comparison with mock-transfected Vero cells. Additionally, a decreased number of ASFV genomes (between 64.87% to 65.83%) and a reduced viral progeny (up to −99.24%) was detected also in pI215L-depleted cells, even though siRNAs targeting I215L transcripts exhibited a moderate gene-silencing efficiency (−23 to −40%). Altogether, these results strongly suggest that ASFV genome replication, viral late transcription and progeny production are mediated thought the ubiquitin pathway, as reported for other human and swine viruses^34^. These findings are schematically illustrated in the proposed working model for ASFV-pI215L presented as Fig. 5.

In summary, our results showed that pI215L plays a key role in ASFV infection, probably by interfering with the ubiquitin machinery and, therefore potentially modulating many viral mechanisms (e.g. transcription, replication and encapsidation) and cellular functions (e.g. antiviral responses, DNA damage responses, apoptosis), raising the hypothesis that an ASFV mutant lacking ORF I215L can be a good candidate for the development of an effective DISC vaccine, a novel vaccination strategy successfully used in other animal viral disease^30^. Indeed, an ASFV I215L-defective mutant is expected to enter host cell and to express the immediate-early genes products, providing enough antigens to induce a protective response in infected pigs and producing a noninfectious progeny that undergo only one cycle of replication. As vaccines, these defective viral mutants are designed to combine the safety and advantages of inactivated vaccines with the immunogenic activity of live viral vaccines, requiring a complementary cell line that expresses pI215L in order to isolate and propagate the ASFV mutant obtained by homologous recombination.

## Material and Methods

### Viruses and cells

The Vero-adapted ASFV isolate Ba71V was used to infect cells and was propagated as described^35^. Infections were carried out at the indicated multiplicities of infection (MOI), and at the end of the adsorption period (1 h), the inoculum was removed and cells were washed twice with serum-free medium. The virus titration was performed on sub-confluent Vero cells grown in 96-well plates inoculated with ten-fold viral dilutions of viral suspensions. Viral infection was assessed by CPE observation and calculated by using the Spearman-Kärber method^36^. Experimental *in vitro* infections were performed using the non-pathogenic ASFV Ba71V strain^37^ and conducted in a BSL-2 facility using BSL-3 work practices.

Vero E6 cells (kidney epithelial cells of African green monkey *Cholorocebus aethiops*) were obtained from the European Cell Culture Collection (ECACC, Salisbury, UK) and maintained as previously reported^38^. All experiments were conducted on actively replicating sub confluent cells.

### Cloning, expression and purification of recombinant ASFV-pI215L

The complete ORF I215L, lacking the stop codon, was PCR-amplified from Ba71V genomic DNA, using the 215Fw and 215Rv primers (Table 1), which include at their 5′ and 3′ ends, NheI and XhoI restriction endonuclease sites to facilitate vector insertion. The PCR reactions were performed as follow: 98 °C for 2 min., 30 cycles at 98 °C for 30 seconds, 65 °C for 30 seconds, 72 °C for 30 seconds plus one extension step at 72 °C for 10 min. After confirmation of correct fragment size by electrophoresis on a 1% agarose gel, the DNA fragments were purified and quantified in the NanoDrop 2000c. Then, these fragments were inserted in a cloning vector (pET24a, Novagen) to add a 6xHis-tag at the tag C-terminal, in order to facilitate the purification step. Two clones were sequenced to avoid mutations generated from Taq polymerase errors. Confirmed plasmids were then transformed into the *E*. *coli* strain BL21(DE3)-pLysS (Novagen) and grown in LB medium (10 g tryptone, 5 g yeast extract, 5 g NaCl, pH 7.2) supplemented with kanamycin (30 μg/ml) plus chloramphenicol (34 μg/ml), at 37 °C, with shaking at 200 rpm, until the OD_600_ reached 0.1–0.2. Induction of protein expression was carried out by adding isopropyl-β-D-1-thiogalactopyranoside (IPTG) at a final concentration of 1 mM during 5 hours. After this step, bacterial cells were harvested by centrifugation (10,000 g for 10 min, 4 °C), and washed with sterile water. The pellet was resuspended in binding buffer (20 mM sodium phosphate, 500 mM NaCl, 20 mM imidazole, pH 7.4) and cells were lysed by a lysis solution (0.2 mg/ml lysozyme, 20 µg/ml DNAse and 1 mM PMSF) and sonicated for 5 × 5 minutes on ice (5 cycles, 70% amplitude). Lysates were then centrifuged at 3000 g for 15 minutes and pellets were discarded. The extracts were thereafter filtered (0.45 µm syringe filter Rotilabo®, CarlRoth) and incubated with Ni Sepharose 6 Fast Flow slurry (GE Healthcare) for 1 hour. The mixture was loaded onto a PD-10 column (GE Healthcare), washed with binding buffer solution (20 mM sodium phosphate, 500 mM NaCl, pH 7.4) containing increasing concentrations of imidazole (40, 60 and 80 mM), and the recombinant pI215L was eluted with an elution buffer (20 mM sodium phosphate, 500 mM NaCl, 500 mM imidazole, pH 7.4). Fractions were collected in low-binding tubes (Maxymum Recovery® TM tubes, Axygen, Corning Life Sciences, Amsterdam, The Netherlands), analyzed by SDS-PAGE and the recombinant pI215L, purified under native conditions, was stored at −80 °C until further use. The three single point mutants (pI215L^C85A^, pI215L^C162A^, pI215L^C189A^) were generated using the QuikChange II XL Site-Directed Mutagenesis Kit (Agilent Technologies), following the manufacturer’s instructions and using the primers indicated in Table 1.

**Table 1:** Primers used in the present study.

Target	Primer designation	Sequence (5′**-**3′)	Target coordinates*	Orientation
ASFV-I215L	215FwE	AGACACCTGATAGAGAACCC	157562–157581	Forward
ASFV-I215L	215FwI	TCCAATGTTCCACCAATACCC	157069–157089	Forward
ASFV-I215L	215RvI	TCATCCATCTCTTCATCCTCCTC	156971–156993	Reverse
Cyclophilin A	CycloFw1	AGACAAGGTTCCAAAGACAGCAG	—	Forward
Cyclophilin A	CycloRev	AGACTGAGTGGTTGGATGGCA	—	Reverse
Cyclophilin A	CycloFw2	TGCCATCCAACCACTCAGTCT	—	Forward
VP72	VP72Fw	ACGGCGCCCTCTAAAGGT	88273–88290	Forward
VP72	VP72Rev	CATGGTCAGCTTCAAACGTTTC	88322–88343	Reverse
VP32	VP32Rev	TCTTTTGTGCAAGCATATACAGCTT	108162–108186	Forward
VP32	VP32Fw	TGCACATCCTCCTTTGAAACAT	108228–108249	Reverse
ASFV-I215L	215Fw	ACTAGCTAGCATGGTTTCCAGGTTTTTAATAGCAGAG	157562–157581	Forward
ASFV-I215L	215Rv	TCCGCTCGAGCTCATCATCCATCTCTTCATCCTC	157069–157089	Reverse
ASFV-I215L	C85AFW	TATTTACCCTGATGGAAGACTAGCTATCTCTATCTTACACGGAGAC	157336–157381	Forward
ASFV-I215L	C85ARV	GTCTCCGTGTAAGATAGAGATAGCTAGTCTTCCATCAGGGTAAATA	157336–157381	Reverse
ASFV-I215L	C162AFW	ATTTTTAAAATATTCTATGTCTTCTGGTGAAGCCTCATCTAATGATTTTTTGACAGTCTTTTTA	157336–157381	Forward
ASFV-I215L	C162ARV	TAAAAAGACTGTCAAAAAATCATTAGATGAGGCTTCACCAGAAGACATAGAATATTTTAAAAAT	157096–157159	Reverse
ASFV-I215L	C189AFW	ATACCCAGTGATGCTTATGAAGATGAAGCTGAAGAAATGGAGGATG	157029–157029	Forward
ASFV-I215L	C189ARV	CATCCTCCATTTCTTCAGCTTCATCTTCATAAGCATCACTGGGTAT	157029–157029	Reverse

*Primer coordinates are relative to Ba71V sequence used has template for primer design.

### ***In vitro*** ubiquitination assay

To determine if the ASFV-pI215L has a catalytic activity similar to an E2 ubiquitin conjugating enzyme, a commercial kit was used (E2-Ubiquitin Conjugation Kit, ab139472, Abcam) and the manufacturer’s instructions were followed. Reactions were performed in a 50 µl mixture containing 5 µl of ubiquitination buffer (50 mM Tris-HCl, pH 7.4, 5 mM MgCl2, 15 μM ZnCl2, 0.3 mM DTT, 0.006% DTT, 2 mM ATP, 10 U creatine phosphokinase, and 10 mM phosphocreatine), 2.5 µl of biotinylated ubiquitin (2.5 µM) or with a mutant biotinylated ubiquitin lacking the seven acceptor lysine residues (Ub^NOK^, Boston Biochem), 2.5 µl of an E1-enzyme (recombinant UBA1 at 100 nM), 5 µl of an E2-enzyme at 2.5 µM (recombinant pI215L or recombinant UbcH5b provided by the kit) and 10 µl of an inorganic pyrophosphatase solution (IPP, 100 U/mL). To investigate whether pI215L-ubiquitin conjugates were mediated by thioester bond formation, samples were incubated at 95 °C with 5% 2-mercaptoethanol (Sigma) during 10 min. Additionally, the reactions were performed in the presence and absence of ATP-Mg^2+^ (2.5 µl at 5 mM) and incubated at 37 °C during 120 min, except when indicated. To further characterize the E2-ubiquitin conjugating activity of pI215L, the assay was also performed at different incubation temperatures (4, 24, 37 and 42 °C) and pH values (4, 7.5, 9 and 14), and different incubation times (1, 5, 15, 30 and 120 min).

Soluble and insoluble protein fractions were prepared from mock infected and Ba71V-infected Vero cells, harvested at 6 and 16 hpi. Initially, cells were washed with PBS and incubated with a buffer containing 50 mM HEPES (pH 7.6), 100 mM NaCl, 2 mM EDTA, 250 mM sucrose, 0.1% Tx-100, supplemented with protease (cOmplete, Mini, EDTA-free from Roche) and phosphatase inhibitors (PhosStop, Roche). After a centrifugation step (10000 × g for 10 min, 4 °C), the supernatant was collected (soluble fraction) and the pellet containing insoluble proteins was lysed in RIPA buffer [25 mM Tris, 150 mM NaCl, 0.5% (v/v) NP40, 0.5% (w/v) sodium deoxycolate, 0.1% (w/v) SDS, pH 8.2] supplemented with protease-inhibitor cocktail (cOmplete, Mini, EDTA-free, Roche) and phosphatase-inhibitor cocktail (PhosStop, Roche). pI215L activity in protein fractions was investigated by incubating the reaction mixtures at 37 °C during 120 min. Reactions were quenched by adding 50 µl of 2x non-reducing gel loading buffer. Reaction products were resolved by SDS-PAGE using 8–16% (w/v) polyacrylamide separating gels and transferred to a 0.2 μm pore diameter nitrocellulose membrane (Whatman Schleider & Schuell) by electroblotting. Finally, membranes were incubated with a streptavidin-HRP antibody (RPM 1231, GE Heathcare, 1:10,000 dilution in 3% TBST-BSA solution) or with the anti-pI215L antibody.

### RNA extraction and cDNA synthesis

Total RNA was extracted using the RNeasy Mini Kit (Qiagen, Courtaboeuf, France) and treated with DNAse I (Qiagen) to remove contaminant genomic DNA. RNA concentrations and purity were measured using a spectrophotometer (NanoDrop 2000c, Thermo Fisher Scientific, Waltham, USA). 200 ng of each RNA sample was reverse transcribed into cDNA using the Transcriptor First Strand cDNA Synthesis Kit (Roche, Basel, Switzerland). The obtained cDNA was diluted (1/20) in ultra-pure water and stored at −20 °C until further use.

### Recombinant plasmids and standard curves

The amplified fragments corresponding to the viral genes (ASFV-I215L, B646L, and CP204L) and the housekeeping gene (Cyclophilin A) were cloned into a pGEM-T Easy Vector System II (Promega, Madison, USA). Each plasmid was used to transform *E*. *coli* DH5α competent cells, followed by an incubation step at 37 °C, under selective antibiotic pressure. Recombinant plasmids were isolated from bacteria using the Roche High Pure Plasmid Isolation Kit (Roche Applied Science, Germany) and quantified by spectrophotometric absorbance (NanoDrop 2000c). Their corresponding copy number was calculated using the equation: pmol (dsDNA) = μg (dsDNA) × 1515/DNA length in pb (pmol = picomoles, dsDNA = double-strand DNA, DNA length in pb = number of base pairs from the amplified fragment; 1 mol = 6,022 × 1023 molecules). Finally, the cloned fragments were amplified by PCR and the sequence confirmed by DNA sequencing. For each amplification plate, a standard calibration curve was obtained for viral genes and for Cyclophilin A to insure the accuracy of the results. Standart curves were plotted by following a previously described protocol^39^.

### Quantitative PCR

Quantification of ASFV-I215L, B646L and CP204L transcripts was performed by qPCR using Maxima SYBR Green PCR Master Mix (Thermo Fisher) according to the manufacturer’s instructions [12.5 µl of master mix, 2.5 µl of forward and reverse primers (50 nM each), 5 µl of Milli-Q water and 2.5 µl of cDNA)]. All qPCR reactions were performed in the Applied Biosystems 7300 Real Time PCR system (Thermo Fisher), and with the following thermal profile: initial denaturation at 95 °C for 10 min followed by 40 cycles of 15 s at 95 °C, 60 °C for 60 s, and a final denaturation step at 65 °C for 5 s with a 20 °C/s ramp rate and subsequent heating of the samples at 95 °C with a ramp rate of 0.1 °C/s. Quantification of ASFV-I215L, B646L, CP204L and Cyclophilin A mRNA levels was determined by the intersection between the fluorescence amplification curve and the threshold line. The crossing point values of each plasmid obtained from different known concentrations were plotted in a standard curve used to determine the copy number of each transcript. The values were determined using the comparative threshold cycle method, which compares the expression of a viral gene normalized to the housekeeping gene (Cyclophilin A). The validation of the housekeeping gene was confirmed using the ANOVA test, whereas the specificity of the qPCR assays was confirmed by the melting curve analysis. The sequences of all primers used in this study are shown in Table 1. To quantify viral gene expression, Vero cells seeded onto 30 mm dishes were infected with a MOI of 1,5. After 1 hour of adsorption, the virus inoculum was washed off with DMEM, and every 2 hours (from 0 to 20 hpi), total RNA extraction was performed from one culture dish. Results were expressed as the mean standard error of the mean and were obtained from three independent experiments performed in different days, to ensure the biological relevance of the results.

### Antibodies

The purified recombinant pI215L was used to produce a mouse polyclonal antiserum. Briefly, young female mice (BALB/c, 4 to 6-week-old) were injected subcutaneously with 100 μg of purified pI215L in a mixture with Freund’s complete adjuvant. Following the primary injection, two booster injections were administered at 2-week intervals. Finally, the total blood was collected 10 days after the second booster injection and sera were aliquoted and stored at −20 °C until further use for immunoblotting and immunofluorescence studies. The specificity of the polyclonal antiserum was tested against purified recombinant ASFV-pI215L and whole infected-cells extracts. The immunostaining of pI215L and ASFV-infected cells was achieved by incubation with two in-house primary antibodies: mouse anti-pI215L [1:10 in PBST (Phosphate Buffered Saline with Tween 20 0.01% v/v), overnight at 4 °C] and swine anti-ASFV polyclonal antibody (1:100, 1 h, RT). Two secondary fluorescent-conjugated antibodies were used as follows: anti-mouse FITC (1:300, sc-2099, Santa Cruz Biotechnology) and anti-swine Texas Red (1:500, ab6775, Abcam). Between each antibody incubation, cells were wash twice with PBS (5 min) and once with PBST (0.1% v/v, 5 min). All incubations were performed in a dark humidified chamber to prevent fluorochrome fading and a mounting medium with DAPI (4′,6-diamidino-2-phenylindole) was used to detect the cell nucleus and viral factories (Vectashield, Vector Laboratories, Peterborough, UK).

For immunoblot analysis, two primary antibodies (anti-pI215L, 1:100; anti-α-tubulin, 1:1250, #2125, Cell Signalling Technology) and two HRP-conjugated secondary antibodies were used (anti-rabbit IgG, 1:10000, 4010-05; anti-mouse IgG, 1:30000, 1010–05; both from SouthernBiotech). All antibody dilutions were performed in blocking solution and incubated according to the manufacturers’ recommendations.

### Immunoblot analysis

Vero cells grown in 30 mm dishes were infected with ASFV-Ba71V isolate (MOI of 5) and when indicated, exposed to cytosine arabinoside (50 µg/ml, AraC; Sigma-Aldrich), after the adsorption period (1 h). Cells were washed twice with PBS and then lysed in ice-cold modified RIPA buffer supplemented with protease-inhibitor cocktail (cOmplete, Mini, EDTA-free, Roche) and phosphatase-inhibitor cocktail (PhosStop, Roche). Clarified whole-cell lysates harvested at 4, 8, 12, 14, 16, 18 and 20 hpi, were then analyzed by western blot technique as previous described^38^, using the above mentioned antibodies. α–Tubulin was used as a loading control.

### Immunofluorescence and microscopy analysis

Vero cells seeded on glass coverslips (1 × 10^5^/cm^2^) were infected with the ASFV Ba71V isolate (MOI of 10). At 4, 8, 12, and 16 hpi, cells were fixed and subsequently processed as previously described^38^. Fluorescence images were acquired using an epifluorescence microscope equipped with a 40x objective (Leica DMR HC model, Wetzlar, Germany) and data sets were acquired with the Adobe Photoshop CS5 software (Adobe Systems, Inc., San Jose, USA).

### siRNA assays

Two double-stranded siRNAs (I215L siRNA_I and I215L siRNA_II; ON-TARGETplus, Thermo Fisher Scientific, USA) targeting different regions of ASFV-I215L mRNA were designed (siDESIGN Center, Thermo Fisher Scientific, USA), based on the full genome sequence of ASFV Ba71V isolate (GenBank/EMBL, accession number: ASU18466). One siRNA against the GAPDH gene (siRNA-GAPDH; Silencer™ GAPDH siRNA human control number 4605; Ambion/Thermo Fisher Scientific) was used as a control. The siRNA sequences targeting ASFV-I215L are shown in Table 2. All siRNAs duplexes were diluted at different final concentrations (10, 50 and 100 nM) in serum-free Opti-MEM (Gibco, Life Technology, Karlsruhe, Germany) and using 8 μl HiPerfect Transfection reagent (Qiagen, Courtaboeuf, France). Mixtures were incubated at room temperature for 20 min to allow the formation of transfection complexes, and thereafter, 100 μl of the transfection solution was incubated with 2 × 10^4^ Vero cells cultured in 500 μl of DMEM supplemented with 10% FBS (24-well plate) for 8 h at 37 °C. One hour after infection, the culture medium was removed and fresh medium was added to allow recovery of the cells. Next, cells were infected with ASFV Ba71V (MOI = 0.1). Then, the virus inoculum was removed one hour after and cells maintained at 37 °C for 72 h. The viability of transfected cells was assessed every 8 hours, until 72 hours, by phase-contrast microscopy. The two different siRNAs were used individually and their antiviral effects were evaluated by the quantification of CP204L and B646L mRNA levels, titration of the ASFV genomes and viral progeny, at 4, 8 and 16 hpi. To ensure high RNA concentrations for qPCR measurements, the siRNA assays were performed in quadruplicated.

**Table 2:** siRNA sequences to knockdown ASFV-I215L transcripts.

Target	siRNA designation	Sequence (5′-3′)	Target coordinates*	Orientation
ASFV-I215L	I215L_I	GUGAAGAAAUGGAGGAUGAUU	565–584	Sense
ASFV-I215L	I215L_II	GCUAAAAGCUACCGUAAAUUU	394–412	Sense

*siRNA coordinates according to the relative position in gene nucleotide sequence (start at position 1, ATG).

### Quantification of ASFV genomes by qPCR

Viral DNA was extracted from Ba71V-infected Vero cells (MOI of 0.1) transfected separately with two siRNAs targeting I215L, at 72 hours post infection (hpi), using the High Pure Viral Nucleic Acid Kit (Roche). The number of viral genomes was determined by quantitative PCR as described by King *et al*.^40^. Mock-infected and infected cells transfected with siRNA-GAPDH were used as controls.

### Statistical analysis

The Kolmogorov-Smirnov test was used to check the normal distribution of the results from the RNAi assays (mRNA expression, ASFV genome copy number and virus titre). Differences between experimental groups were assessed using the non-parametric Wilcoxon-Mann-Whitney test, because a normal distribution was not obatained. *p-*values less than 0.05 were considered significant and the GraphPad Prism software (version 7.02) was used to perform statistical analysis.

## Electronic supplementary material

Supplementary Information
