# Supplementary material for: African swine fever virus encodes for an E2-ubiquitin conjugating enzyme that is mono- and di-ubiquitinated and required for viral replication cycle
Source: Sci Rep. 2018 Feb 22;8:3471. doi: 10.1038/s41598-018-21872-2 (PMC5823848; doi:10.1038/s41598-018-21872-2)

African swine fever virus encodes for an E2-ubiquitin conjugating enzyme that is mono- and di-ubiquitinated and required for viral replication cycle

Ferdinando B. Freitas, Gonçalo Frouco, Carlos Martins, Fernando Ferreira

Supplemental Materials

SUPPLEMENTARY FIGURE S1 - Binding parameteres of ASFV E2-ubiquitin conjugating enzyme**,** full-length blots.

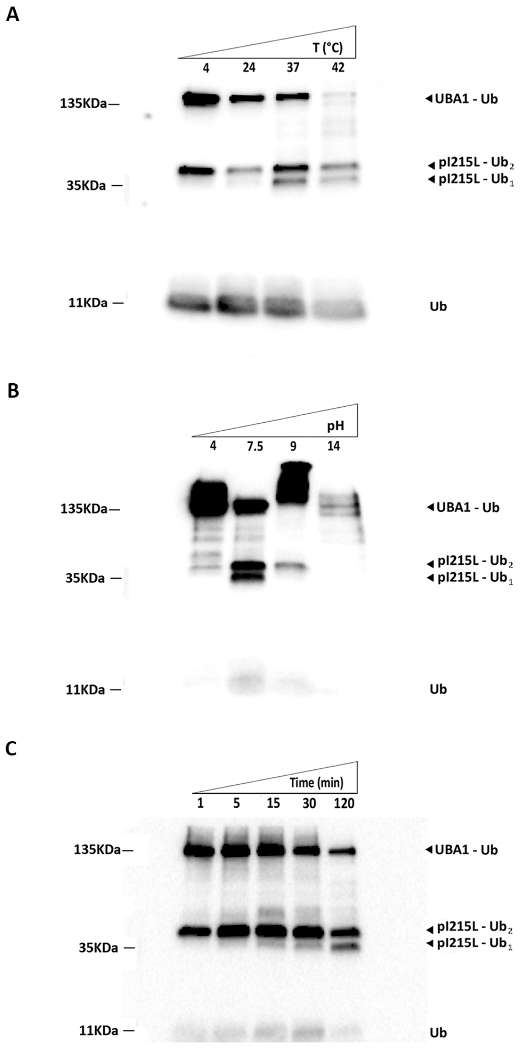

Supplement: Supplementary file 1 — Supplementary Information [file 41598_2018_21872_MOESM1_ESM.pdf]
